# *Mothers In Motion* Program: Implementation Process and Acceptability by Community

**DOI:** 10.3390/nu15122739

**Published:** 2023-06-14

**Authors:** Mei-Wei Chang, Lisa K. Militello, Janna D. Stephens

**Affiliations:** The Ohio State University College of Nursing, 1585 Neil Avenue, Columbus, OH 43210, USA; militello.14@osu.edu (L.K.M.); stephens.653@osu.edu (J.D.S.)

**Keywords:** implementation process, FRAME-IS, lifestyle behavior, compatibility, complexity

## Abstract

Background: Implementing lifestyle behavior programs in real-world settings challenges researchers. The Special Supplemental Nutrition Program for Women, Infants, and Children (*WIC)* has implemented and sustained *Mothers In Motion* (*MIM*)’s client videos for clients to promote healthy lifestyle behaviors, and train-the-trainer videos, for personnel to enhance motivational interviewing techniques since 2015 and 2016, respectively. This paper describes the implementation processes and the results of client video implementation acceptability by WIC personnel. Methods: To document the implementation process, we applied the Framework for Adaptation and Modifications to Evidence-Based Implementation Strategies (FRAME-IS). To evaluate implementation acceptability, we conducted semi-structured interviews with 15 WIC personnel. A qualitative analysis was conducted to identify the common themes. Results: The facilitators for client video implementation were the inclusion of the target audience and family members addressing daily challenges, easy implementation, and compatibility with daily practice. While videos online facilitated implementation, videos in DVD format could challenge implementation. Conclusions: Future lifestyle intervention programs aimed for future implementation in community settings may consider the inclusion of the target audience and their family members and take into consideration easy implementation and compatibility.

## 1. Introduction

Although low-income mothers with young children desire to eat healthier and be more physically active (healthier lifestyle behavior), many factors impede their ability to engage in healthy lifestyle behavior [1,2,3]. For example, living stressful lives increases emotional eating and unhealthy eating and decreases physical activity. Also, limited or no access to free stress management programs that address daily challenges to promote mental health is an issue. Moreover, there is a lack of access to culturally sensitive intervention programs that can be easily related to the target audience and include practical strategies to be easily implemented in their daily lives to make positive lifestyle behavior changes [1,2,3,4]. Promoting healthy lifestyle behaviors among these mothers is critical because they are gatekeepers of their young children and their behaviors influence their children’s eating and physical activity patterns [3,5]. Thus, having access to free lifestyle intervention programs addressing the needs of these low-income mothers is imperative.

An excellent setting to broadly reach low-income mothers with young children to promote healthy lifestyle behavior is through the Special Supplemental Nutrition Program for Women, Infants, and Children (WIC). WIC is one of the largest federally funded nutrition programs in the United States and served nearly 6.3 million enrollees in 2020 [6]. One in two American children were WIC babies [7]. WIC provides nutrition consultation and food vouchers and makes referrals. To receive a food voucher, WIC clients are required to complete at least one nutrition educational training every three months through (for example) a secured and password-protective website that is shared by WIC nationwide. Once clients complete a training module, local WIC offices are notified in their system. The website included predominately written text and links from credible websites about (for example) healthier lifestyle behavior and infant feeding. WIC dietitians are committed to the promotion of healthy lifestyle behavior. However, they face time constraints that limit their ability to provide adequate healthy eating or physical activity counseling, or both, to their clients. Although WIC stakeholders are aware of and understand the negative impact of stress on their clients’ lifestyle behaviors, WIC dietitians are not trained in delivering stress management. More importantly, there are limited free stress management programs that address the daily challenges of WIC mothers.

Implementing and sustaining a lifestyle intervention program in real-world settings remains significantly challenging, especially after the researcher-led program ends [8,9,10,11]. Implementing and sustaining a lifestyle intervention program in community settings is even more challenging because of the lack of reimbursement by public or private insurance companies [12]. In addition to the above-mentioned challenges, a known barrier to implementing lifestyle interventions is the selection of appropriate implementation strategies for a given intervention [13]. Up-to-date descriptions of successful lifestyle intervention implementation strategies used have been limited in clinical settings [14], leaving successful implementation strategies in community settings unclear. Another barrier to implementation is the lack of clear documentation of the implementation process [15]. Therefore, the objectives of this paper were to describe the implementation process and to present the results of the client video implementation (described in detail below) acceptability by WIC personnel.

*Mothers In Motion* (*MIM*, Principal Investigator [PI]: Chang) was a community-based randomized controlled lifestyle behavior intervention program. *MIM* translated Diabetes Prevention Program (an evidence-based program) into a real-world setting [16]. *MIM* aims to prevent further weight gain in overweight or obese mothers with young children through the promotion of stress management, healthy eating, and physical activity. *MIM* participants were recruited from WIC. The intervention was designed with future implementation in WIC settings in mind. The 16-week intervention had two components: unscripted culturally sensitive videos, hereafter, client videos (a total of 10 modules, 20 min per module) via DVD, and peer support group teleconferences (a total of 10 sessions, 30 min per session). The client videos featured overweight or obese WIC mothers and their family members, especially young children. They provided testimonies of daily challenges associated with stress management, healthy eating, and physical activity. They also demonstrated easy and practical strategies that could be easily implemented in daily life to make positive changes. The peer support group teleconferences were led by peer educators and WIC dietitians. They attended a two-day in-person motivational interview training and applied learned techniques to lead the teleconference [16]. *MIM* intervention helped reduce dietary fat intake [17], alleviated stress and depressive symptoms [18], and increased physical activity [19]. However, the intervention did not affect body weight [20].

## 2. Methods

In this section, we will first describe the implementation process by applying the Framework for Adaptation and Modifications to Evidence-Based Implementation Strategies (FRAME-IS), followed by presenting the results of implementation acceptability. Table 1 summarizes the implementation process by applying FRAME-IS with seven modules.

### Implementation Process

Module 1. A Brief Description of the *MIM*, Implementation Strategies Used, and Modifications

A brief description of *MIM.* We have briefly described *MIM* (see above). Implementation Strategies and Modifications. We applied The Expert Recommendations for Implementation Change (ERIC) to describe the implementation strategies: share success stories, work closely with informal local opinion leaders, work closely with champions, involve WIC clients and family members, and develop an implementation blueprint.

Share Success Stories. During the *MIM* intervention phase, the PI sent a monthly email to WIC stakeholders and collaborating sites to share success stories. These stories were taken from peer support group teleconferences where intervention participants shared the positive impact of *MIM* on their own and their family members’ lives, especially young children. Also, many intervention participants shared the benefits of joining the *MIM* with WIC dietitians and nurses during their WIC appointment.

Work Closely with Informal Local Opinion Leaders. During the intervention phase, WIC managers at our collaborating sites became informal local opinion leaders after hearing *MIM* success stories from their clients, dietitians, and nurses. Consequently, these leaders expressed a strong interest in adopting and implementing the *MIM* in the WIC setting. They brainstormed with the PI about ways to use the client videos and recommended strategies to successfully work with stakeholders at the State of Michigan WIC to implement *MIM.*

Working Closely with Champions. WIC dietitians who led the peer support group teleconferences (hereafter, moderators) become champions because they repeatedly heard success stories when leading the peer support group teleconferences. They shared the success stories with their managers and colleagues. One of the moderators was a member of the Michigan WIC Nutrition Education Group who made recommendations to the State of Michigan WIC on procedure and policy change. During one of the Education Group meetings, the moderator shared success stories with the group and showed a preview of the client videos prepared by the PI.

Involve WIC clients and family members. WIC personnel at all levels equivocally appreciated the unscripted client videos featuring WIC clients and their family members, especially young children. They said that the stories in the videos were real because they heard them frequently in their daily practice. They also agreed that the strategies used for stress management, healthy eating, and physical activity were simple and practical, and believed that their clients could easily relate to the characters in the videos and apply these strategies to make positive changes. The above-mentioned WIC perceptions were consistent with our *MIM* acceptability evaluation by the intervention participants [20].

Develop Implementation Blueprint. WIC stakeholders took the lead to make implementation plans. For example, they secured a USDA grant to support the implementation. They presented success stories and a preview of client videos to their colleagues in other states. The PI attended the national WIC conference to give a workshop for client videos and train-the-trainer videos (described below).

Modifications and Reasons for Modifications. Stakeholders did not perceive it to be feasible to distribute client videos in DVD format and decided to put these videos on a WIC nationwide shared website (described above) to broadly reach WIC clients. Similarly, stakeholders did not perceive it to be feasible to conduct a two-day in-person motivational interviewing training session due to the cost and time. They suggested converting in-person training to online training using the train-the-trainer model to save training costs and time.

Module 2. What was Modified?

Client videos. The PI added healthy eating content to four stress management modules to meet USDA requirements, as per the stakeholders’ request. She also added content to help clients choose appropriate strategies to accomplish their goals as per the *MIM* intervention participants’ request. When the videos were available via the secure and password-protected WIC website, WIC clients nationwide could review any modules at their convenience and as many modules as they want to meet their needs instead of completing ten modules in 16 weeks as per the *MIM* intervention protocols. Train-the-trainer videos (for WIC personnel). The PI created ten train-the-trainer video modules (1 h per module). These videos were available through the Michigan WIC website. The Michigan WIC personnel could log in to the website and review the module(s) to meet their needs at their convenience time and location.

Module 3. What is the Nature of the Content, Evaluation, or Training Modification?

Client videos. The PI added healthy eating with a focus on increasing fruit and vegetable intake and reducing junk food intake in young children to the stress management modules. She also added additional content with an emphasis on applying and selecting learned skills to accomplish a goal. The newly added content was reviewed by more than 20 WIC mothers in two rounds. The PI used their feedback to finalize the videos. Consequently, the client videos were extended from 20 to 30 min per module. Train-the-trainer videos. Before converting in-person motivational interviewing training to online training, the PI conducted a needs assessment. She met with informal local opinion leaders, champions, and WIC personnel. They expressed the need to include nonverbal cues observation, especially tone of voice when interacting with clients. The scenarios included in the training videos must be relevant to WIC daily practice. The application of techniques must be easy and practical to apply to daily practice. Moreover, they suggested the inclusion of the benefits of applying motivational interviews to personal life (in addition to benefiting clients) and clarification of the misconceptions of applying motivational interviews. The PI created ten modules that included PowerPoint slides and selected mini videos for practicing motivational interviewing techniques and nonverbal cues. The mini videos featured WIC mothers’ testimonies about daily challenges in making positive change. The draft contents of each training module were pilot tested to ensure the contents met the needs of WIC. The pilot testing was conducted in person and included seven WIC dietitians (one trainer and six trainees). Then, the PI used their feedback to finalize the training contents. Since client videos were well-received by the WIC at all levels, the PI decided to include WIC dieticians in the training video: one trainer with expertise in motivational interviewing training (the same trainer in the pilot testing) and six trainees who did not participate in the pilot testing. These trainees had no or some experience in applying motivational interviewing techniques. Filming was done in a WIC conference room. The training videos were totally unscripted to increase the relatability of the characters in the videos and buy-in. Each draft module (a total of ten modules) was reviewed by at least four WIC dietitians. They viewed the video first and then applied the learned skills to daily practice for one week prior to providing feedback for revision. The PI used their feedback to finalize the training modules.

Module 4. What is the Goal and the Level of the Rationale for Modification?

The short-term goal and rationale for the modifications to both client and train-the-trainer videos were to increase the acceptability and feasibility of *MIM* implementation and sustainability. The long-term goal was to broadly reach WIC clients and personnel for a broad impact.

Module 5. When is modification initiated and Is modification planned?

The modifications for both client and the train-the-trainer videos were initiated by stakeholders during year four of the five-year *MIM* intervention project. The modifications were detailed and planned and were a joint effort reflecting principles of community-based participatory research.

Module 6. Who participated in the decisions to modify?

Client videos. The PI who developed and conducted *MIM* led decisions on modifications. She incorporated suggestions from stakeholders and *MIM* intervention participants.

Train-the-Trainer-videos. The PI led the decision on converting in-person to online training. Also, WIC stakeholders and personnel at all levels (see Module 3 described above for details) participated in the decision on content modifications.

Module 7. How Widespread Was the modification?

Prior to implementation, stakeholders created a benchmark to evaluate the success of implementation. Client videos. The revised client videos have been implemented and sustained by WIC nationwide since 2015 because WIC client evaluation exceeds the benchmark. Train-the-trainer videos. These training videos have been implemented and sustained by Michigan WIC since 2016; all WIC personnel such as dietitians, nutritionists, and nurses are required to attend the online training. Again, WIC personnel evaluated the videos favorably, which exceeds the WIC benchmark.

ACCESSING ACCEPTABILITY OF IMPLEMENTATION OF CLIENT VIDEOS VIA DVD FORMAT AND ONLINE

Our *MIM* collaborating sites decided to implement client videos via DVD format prior to the online version available because they perceived these videos to be helpful to their clients. Several months after implementation, these videos became available online.

Participants, setting, and procedure. Participants were WIC personnel (managers, dietitians, and nurses) employed by Michigan WIC who had been involved in the client video implementation via DVD format for at least six months. They were recruited via email to evaluate the acceptability of the client video implementation. Those who were interested in participating received a consent form via email and were given an opportunity to ask questions prior to the interview. This study was exempted from signed consent form requirements. A trained interviewer used a semi-structured interview guide to conduct 15 individual phone interviews, which reached content satisfaction. Each phone interview lasted about 15 min and was audio recorded with the participants’ permission. The study procedure was approved by The Ohio State University Institutional Review Board (IRB #2016B0030).

Qualitative analysis. The audio recordings were transcribed verbatim. The transcription was reviewed for accuracy. The PI created a codebook. A deductive process was applied to identify common themes among the participant’s comments. The PI and a research assistant independently performed the content analysis, then compared their codes and discussed discrepancies until a consensus was reached.

## 3. Results

### 3.1. Demographics

Of 15 participants, 4 (26.7%) were WIC managers and 11 (73.3%) were WIC dietitians (*n* = 6) and nurses (*n* = 5); the mean working years for WIC was 9.9 years (SD = 5.7 years). The mean age of the study participants was 43.3 years (SD = 12.3). The sample was predominately Non-Hispanic Whites (12/15, 80.0%); 2 were Asian Americans (13.3%); and 1 (6.7%) was mixed race. All participants had at least a college education; 13 (86.7%) worked full-time and 2 (13.3%) worked part-time.

### 3.2. Common Themes

Implementation Plans. When asked about the first step of implementing the videos in DVD format, WIC managers consistently said that they had their staff (dietitians, nurses, or nutritionists) watch at least one module. They distributed DVDs to clients to take home to watch; one WIC office had clients watch a module while waiting for or after their appointment. They also followed up with clients who received DVDs to watch at home to verify whether they viewed the videos.

Barriers to implementation. Staff shortages could limit the implementation of client videos in DVD format. To issue a food voucher to clients, the WIC needed to follow up with clients to verify whether they viewed at least one video module in DVD format. This process was perceived as a challenge. “*The biggest challenge is that when the clients select to view it* (client videos) *for their education, they’re committing to that 3 months before it’s actually due. So we send the videos home with them…our population is often moving, and you know, lots of stressors going on. When the education is due, sometimes they remember that they’ve signed up to do it and they can’t find the DVDs*”. “*There were some challenges of client of either having the interest to take them or saying they didn’t have a DVD player or then following through and doing them and calling back*”. When the client videos were put online (at the same time the minimum DVD was left for distribution), participants were concerned some clients would not have access to these videos because of a lack of access to the internet.

Inclusion of target audience and family members to address daily challenges. Participants unequivocally said that they strongly supported implementation, because the video featured WIC mothers and their family members that addressed daily challenges for making positive changes. “*There is a lot of in-depth things discussed that probably all the moms are struggling with to some extent with so many of our moms having issues struggling with weight, making healthy meals*”. “*We don’t have time to talk about grocery shopping, often times you don’t have time to talk about stress. So the videos are something like that we could give the mom that would allow us to continue the education, on her time outside the clinic*”. “*The client will pick like one of the lessons* (modules) *to do for their nutrition education,… they end up watching more than one, so that’s being cool to see*”. “*They really appreciated it* (client videos) *and they felt more confident and implemented some of the suggestions in the videos at home with their kids*”. “*Meal planning, that has been the most popular one… so they* (WIC clients) *said to me ‘I’ve started meal planning after I watched that one and it’s really been a positive impact on the kids too.’ They’re eating healthier and they are kind of doing thing differently as a family, so it impacts more than just the clients that we see, it’s all the other kids in the family, and both parents*”.

Easy implementation. Participants said that implementation was easy via the DVD format and especially online. “It’ll be easy for the client to just log in and watch it that way…just seeing that as an option will get more people to do it”. “*They (client videos) are online now, which is going to be more helpful I think versus the DVD thing... that’s going to be easier way to promote it, especially for moms who already doing the nutrition education online*”.

Compatibility. Participants consistently said that the implementation of the client videos was compatible with their daily practice when distributing the videos online or in DVD format to watch at home. They introduced the videos to clients during their appointment and encouraged them to watch them. “*We tried to just give it as an option*... *we usually say something like ‘you can do it online, you can come to the office, or you can do the DVD too*”. “*I* (WIC mom) *don’t have time to do these things; I don’t have money to grocery shop healthy…then I* (WIC personal) *go on to say well we do have some DVDs that can help. They’re other moms* (WIC moms in the videos) *who kind of talk about their struggles and how to overcome things*”. However, WIC personnel said having their clients watch videos during WIC appointments was not compatible with their daily practice. “*Our clients come in for the WIC appointment and itself is like 2-hour long and it’s hard to add the video on the top of the 2-hour. Some of them come in with like 2 or 3 kids, some even 4 or 5 children, they’re running around, crying, or hungry. It’s just not doable for them*”. “*There is no way that these moms would sit and watch with the interruption with the kids in the room*”.

Recommendation of the client videos to other WICs. WIC personnel consistently said that they strongly supported implementation and would recommend other WIC personnel to encourage their clients to view the videos. This is because the videos included real WIC mothers and their family members providing testimonies and demonstrating practical tips to overcome daily challenges to make positive changes. Also, the videos included stress management, which was a high-need topic for many WIC clients.

## 4. Discussion

This paper described the implementation process and presented the results of implementation acceptability by WIC managers, dietitians, and nurses. Based on conversations with the WIC at all levels while planning the implementation and the results of our qualitative interview, we learned that unscripted videos played a key role in the success of implementation. Indeed, the unscripted videos were requested by WIC moms who participated in our focus group discussions (before *MIM* development) [1]. These mothers recommended the inclusion of their peers and family members, especially young children, in the videos to show how they went through daily challenges in making positive changes. Identifying WIC moms to be in the videos and creating unscripted videos was time-consuming and challenging but worth pursuing. This is because the target audience became motivated by relating characters in the videos and became empowered by seeing the success of their peers. The PI used the same approach to create the train-the-trainer videos, which were well received by the WIC at all levels.

We also learned the importance of having supportive leaders from the community (stakeholders and informal local opinion leaders), supportive climate and culture, and existing technology infrastructure. The client and train-the-trainer videos were easy to put online to broadly reach the target audience because the WIC already has an existing technology infrastructure, and the target audience knows how to navigate the websites.

Other factors contributed to our success. Interpersonal relationship networks played a role in furthering the dissemination [21]. The PI has collaborated with the Michigan WIC since 2003 to conduct numerous preliminary studies to design and pilot *MIM* [22] followed by conducting a large-scale assessment of *MIM* [16]. Michigan WICs at both state and local levels were actively involved in many aspects of the large-scale *MIM* (e.g., assisting in recruitment and project evaluation, reviewing intervention videos, and disseminating study findings and videos) since it was founded in 2011.

## 5. Limitations to the Study

Our acceptability evaluation was focused on WIC managers, dietitians, and nurses at our collaborating sites. We did not interview WIC clients, because the client videos were already well-received by *MIM* intervention participants. Also, our evaluation only focused on client videos. The fact that both client and WIC staff train-the-trainer videos have been sustained since 2015 and 2016, respectively, demonstrates the value of these videos to WIC.

## 6. Conclusions

Implementation of a lifestyle behavior intervention program in community settings challenges researchers. Our client and train-the-trainer videos have been successfully implemented and sustained in the WIC setting. This is because we utilized numerous strategies, for example, unscripted videos, including the target audience and their family members, existing technology infrastructures, easy implementation, and compatibility with daily practice.

## Figures and Tables

**Table 1:** A Description of the Implementation Process Using the Framework for Adaptation and Modifications to Evidence-Based Implementation Strategies (FRAME-IS).

FRAME-IS Module and Sub-Component	Completion
Module 1	
The innovation being implemented is:	*Mothers In Motion:* Client videos Train-the-trainer videos for WIC personnel
The implementation strategy used	Share success stories, work closely with informal local opinion leaders and champions, involve WIC clients and family members, and develop implementation blueprint
The modification(s) being made are:	Client videos. Deliver online instead of DVD format Train-the-trainer videos. Deliver online rather than 2-day in-person training
The reasons(s) for modification(s) are:	Client videos. To broadly reach WIC clients Train-the-trainer videos. To save training cost and time
Module 2	
What is modified?	Client videos. Content and delivery Train-the-trainer videos. Content and delivery
Module 3	
What is the nature of the content, evaluation, or training modification	Client videos. Add nutritional contents to stress management modules and selection of appropriate strategies for accomplishing goal Train-the-trainer videos. Develop contents that were relevant to daily practice and emphasized practical application
Module 4	
What is the goal?	Client videos. Increase acceptability, feasibility, and sustainability Train-the-trainer videos. Increase acceptability, feasibility, and sustainability
What is the level of the rational for the modification	Client videos. To meet the needs of WIC clients Train-the-trainer videos. To meet the needs of WIC personnel
Module 5	
When is the modification initiated?	Client videos. Year 4 of the 5-year *MIM* program Train-the-trainer videos. Year 4 of the 5-year *MIM* program
Is the modification planned	Client videos. Planned and proactive Train-the-trainer videos. Planned and proactive
Module 6	
Who participated in the decision to modify?	Client videos. Researcher, WIC stakeholders and clients Train-the-trainer videos. Researcher, WIC stakeholders, managers, dietitians, and nurses
OPTIONAL: Who makes the ultimate decision?	Researcher
Module 7	
How widespread is the modification?	Client videos. Implement and sustain by WIC nationwide Train-the-trainer videos. Implement and sustain by State of Michigan WIC

## Data Availability

Data are not available for other researchers, because we are actively analyzing data to answer our proposed research questions.
